# Tetanus: Recognition and Management

**DOI:** 10.1016/S1473-3099(25)00292-0

**Published:** 2025-06-18

**Authors:** Raghav Sudarshan, Ana Ria Sayo, David Roman Renner, Sophia de Saram, Gauri Godbole, Clare Warrell, Ha Thi Hai Duong, C Louise Thwaites, Arpan R. Mehta, Charles Coughlan

**Affiliations:** 1 Peterborough City Hospital, North West Anglia NHS Foundation Trust, Peterborough, UK; 2 Department of Infectious Diseases, San Lazaro Hospital, Manila, Philippines; 3 Department of Neurology, University of Utah, Utah, USA; 4 Kenya Moi Teaching and Referral Hospital, Eldoret, Kenya; 5 University College London NHS Foundation Trust, London, UK; 6 UK Health Security Agency, London, UK; 7 Imported Fever Service, Rare and Imported Pathogen Laboratory, Porton Down, Salisbury, UK; 8 London School of Hygiene and Tropical Medicine, London, UK; 9 Hospital for Tropical Diseases, Ho Chi Minh City, Vietnam; 10 Oxford University Clinical Research Unit, Ho Chi Minh City, Vietnam; 11 Centre for Tropical Medicine and Global Health, University of Oxford, Oxford, UK; 12 Centre for Clinical Brain Sciences, University of Edinburgh, Edinburgh, UK; 13 MRC Protein Phosphorylation and Ubiquitylation Unit, School of Life Sciences, University of Dundee, Dundee, UK

## Abstract

Currently a rarity in high-income countries, tetanus is a diagnosis not to miss. Deaths from tetanus fell by almost 90% between 1990 and 2019, largely reflecting the success of the World Health Organization’s Maternal and Neonatal Tetanus elimination campaign. However, deaths amongst children and adults have plateaued, and tetanus remains an important vaccine-preventable cause of morbidity and mortality, notably in South Asia, Southeast Asia, and Sub-Saharan Africa. Tetanus results from infections with spore-forming *Clostridium tetani* bacteria, usually acquired via contaminated wounds and burns. *C. tetani* releases a potent neurotoxin, causing muscle spasms, rigidity, and dysautonomia. Important complications include laryngeal spasm, leading to airway obstruction and respiratory arrest; nosocomial infections; and sequelae of prolonged immobility. Tetanus is a clinical diagnosis, but microbiological tests may serve as useful adjuncts. Treatment is multifaceted, requiring source control, antibiotic therapy, and antitoxin administration. With prolonged, quality intensive care, many patients survive with good functional outcome. However, due to challenges in leveraging routinely-collected healthcare data and performing high-quality trials in resource-constrained settings, several key questions remain unanswered and optimal treatment strategies are contested. In this review, we provide a state-of-the-art summary of global tetanus epidemiology; its clinical features and differential diagnosis; principles of management; and prognosis.

## Introduction

Tetanus is a devastating neurological infection that remains a major cause of global morbidity and mortality despite a widely available, highly-effective vaccine, and improved access to antitoxin and intensive care. It is caused by *Clostridium tetani*, a ubiquitous, spore-forming, Gram-positive obligate anaerobe. *C. tetani* produces a potent neurotoxin. Tetanus neuro-toxin (TeNT) is transported retrogradely to the central nervous system (CNS), where it blocks gamma amino-butyric acid (GABA) and glycine release from inhibitory interneurons. The resulting disinhibition provokes unopposed motor neuron activity, giving rise to the clinical hallmarks of tetanus: muscle spasms and rigidity.^1–3^

There are four forms of tetanus - neonatal, localised, cephalic, and generalised disease. All share core clinical features. Neonatal tetanus arises in the first 28 days of life; it carries a poor prognosis, and each case reflects an avoidable cascade of failed care, encompassing incomplete maternal vaccination, weak antenatal health systems, and unclean perinatal practices. It is closely intertwined with ‘maternal’ tetanus, which occurs in adolescent or adult women during pregnancy, or in the six weeks after miscarriage, stillbirth, termination or delivery.^4^ These topics are discussed in detail elsewhere.^5^ Localised tetanus, typically affecting a single, injured limb, has a favourable prognosis, but may generalise without prompt treatment. Cephalic tetanus, a variant of localised tetanus circumscribed to the head and neck, is characterised by trismus, dysphagia, and one or more cranial nerve palsies; diagnosis is often delayed and outcomes poor. Generalised tetanus manifests with widespread, painful spasms, trismus, and dysphagia. Patients with generalised disease frequently require mechanical ventilation (MV) and prolonged stays in an intensive care unit (ICU). In many high-burden settings, this level of care is unavailable or financially catastrophic.

## Epidemiology

**Figure 1:**
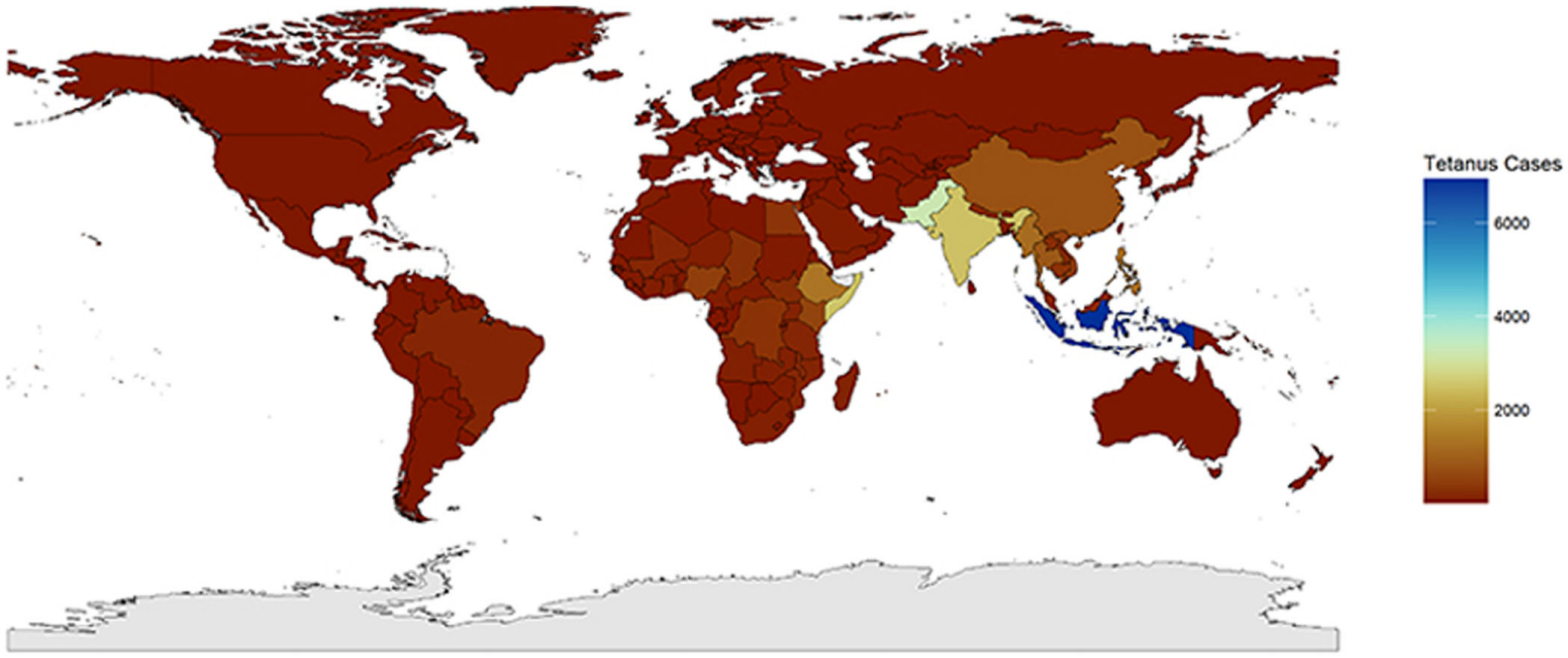
Global Tetanus Cases in patients aged ≥20 in 2021 Legend: New cases of tetanus in adults aged ≥20 in 2021. Differing case burden may reflect unequal reporting and can therefore under-represent certain regions. Data from Global Burden of Disease Collaborative Network. Global Burden of Disease Study 2021 (GBD 2021). Seattle, United States: Institute for Health Metrics and Evaluation (IHME), 2024. GBD Compare. Seattle, WA: IHME, University of Washington, 2015. Available from http://vizhub.healthdata.org/gbd-compare (Accessed 19 October 2024).

Fortunately, high vaccine uptake has rendered tetanus rare across much of the world. Between 1990 and 2019, the global burden of disability-adjusted life years and deaths associated with tetanus fell by 93% and 88%, respectively.^6^ This reduction in mortality has been especially marked amongst neonates and pregnant women; by 2018, the World Health Organization’s (WHO) Maternal and Neonatal Tetanus Elimination (MNTE) initiative led to elimination of MNT as a public health problem in all but 14 countries.^4^ Nonetheless, data are disputed; the decline in tetanus cases has been less marked in children and adults,^6^ and global trends mask differences in disease burden across income settings (Figure 1).

In our view, the reported burden of non-neonatal tetanus is likely to be a substantial underestimate. This is reflected in inconsistent estimates of incidence and mortality. 21,830 cases of tetanus were reported to WHO in 2023.^7^ However, disease modelling suggests a far higher burden of non-neonatal tetanus than reported,^8^ and experts estimate global mortality from tetanus at 30-50,000 deaths/year.^9^ This discrepancy reflects systemic weaknesses in case reporting (many high-burden countries do not classify tetanus as a notifiable disease) and epidemiological surveys that focus on mortality while overlooking critical care survivors. A large Ugandan study recently reported an incidence of 3.43 cases per 100,000 population per year^10^; this figure, though high, is probably representative of many countries with similar vaccination coverage.

Over 90% of tetanus deaths occur in South/South East Asia, and sub-Saharan Africa.^6^ Nevertheless, *C. tetani* is distributed worldwide, with 32-92 cases diagnosed each year in the European Union/European Economic Area^11^; 17-33 in the USA^12^; and 4-11 in the UK.^13^ Despite being notifiable, a study of hospital records from 2001-2014 suggested that cases in England were under-reported by 88%.^14^ There appears to be seasonality in Europe, with highest incidence in July, and a second peak in October.^11^ This likely reflects increased outdoor activity, which carries risks of skin breaks and wound contamination.

WHO has recommended universal childhood tetanus toxoid vaccination since 1974.^15^ Serial vaccination is key. This induces a potent IgG response; antibodies are transmitted transplacentally and vaccinating girls therefore prevents neonatal tetanus. Primary immunisation with a three-dose regimen provides near-total protection against clinical disease, maintained for at least 5 years.^16^ Children in Britain receive a primary course at 2, 3, and 4 months, with pre-school and teenage booster doses. This five-dose regimen is probably sufficient to confer lifelong immunity, though WHO recommends six,^17^ and global booster schedules vary.^18,19^

As of 2022, 92% of children in the UK were fully vaccinated, similar to other high-income anglophone countries.^20,21^ However, there is no herd immunity, and with growing concerns about parental vaccine hesitancy,^22^ and interruptions during the COVID-19 pandemic,^23^ inquiring about childhood vaccination status is crucial. Conflict and natural disasters can also affect vaccination coverage, even in countries with historically robust programmes.^24,25^

Major risk factors for tetanus include advanced age, injecting drug use, and diabetes mellitus. Sporadic tetanus outbreaks in the UK have been linked to contaminated heroin.^26^ Older individuals are at particular risk; they are more likely to have been born before the rollout of universal childhood vaccination, and vaccine-induced antibody levels diminish with age.^27,28^ Cases are more frequent in men, reflecting unsafe circumcision practices,^29^ and lower vaccination rates due to WHO’s maternal vaccination focus.^30^

## Pathophysiology

**Figure 2:**
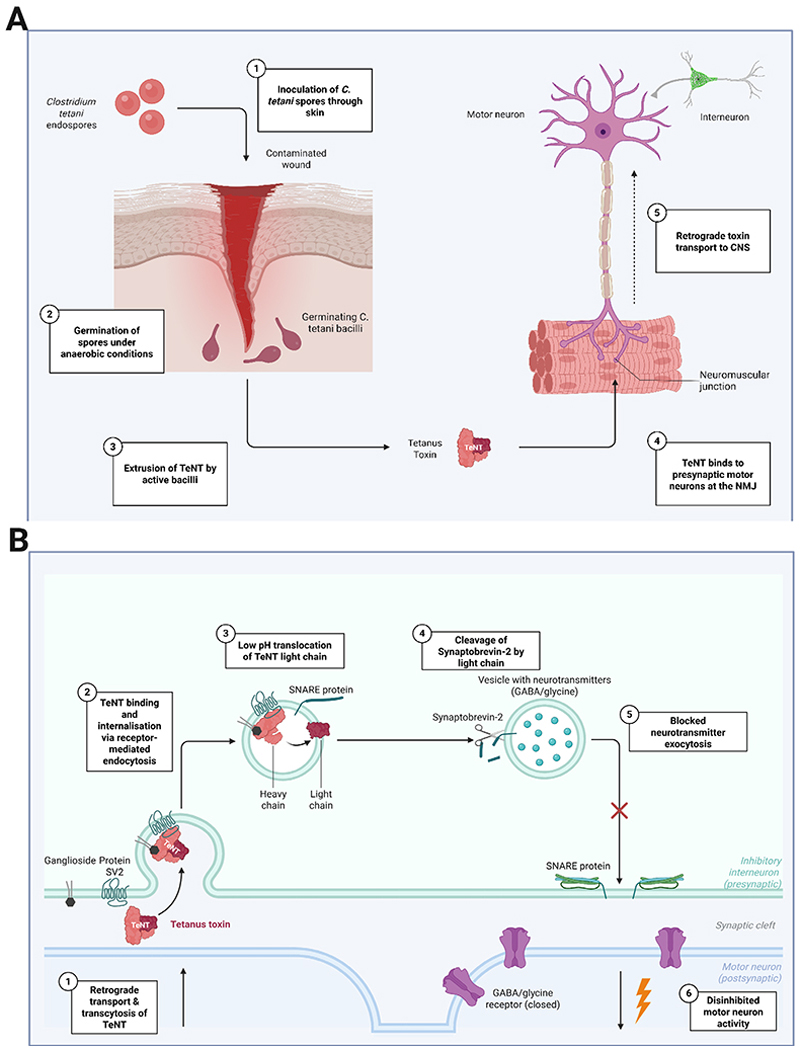
(A) - Pathophysiology of Tetanus from spore inoculation through toxin production and transport to CNS inhibitory interneurons. (B) Toxin pathophysiology in CNS interneurons leading to disinhibition of motor neurons Legend: Figure 2A: *C. tetani* spores germinate into bacilli in anaerobic conditions, typically in the setting of tissue necrosis. Tetanus neurotoxin (TeNT), a 150kDa zinc-dependent metalloproteinase composed of a heavy and catalytic light chain linked by a single disulphide bond, is released by bacterial autolysis. TeNT binds to presynaptic motor neurons at the neuromuscular junction (NMJ) and is transported by retrograde axonal transport and transcytosis to upstream inhibitory interneurons in the central nervous system (CNS). NMJ = Neuromuscular junction. Figure 2B: TeNT enters CNS inhibitory interneurons by receptor-mediated endocytosis. Under acidic vesicular conditions, the light chain is translocated across the endosomal membrane, and the disulphide bond subsequently reduced by cytosolic thioredoxin-thioredoxin reductase systems, freeing the enzyme. Within the cytosol, this acts to cleave synaptobrevin-2, also known as Vesicle-associated membrane protein 2 (VAMP2), on synaptic vesicles. Inhibitory neurotransmitter exocytosis is therefore blocked, leading to disinhibited downstream motor activity. Figure created with Biorender.com.

The defining features of tetanus—muscular rigidity, spasms, and autonomic instability—stem directly from tetanus toxin (TeNT). Previously termed tetanospasmin, TeNT targets inhibitory interneurons in the CNS. Understanding the toxin mechanism (Figure 2) is essential for interpreting symptoms, anticipating complications, and guiding treatment.

*C. tetani* spores, ubiquitous in soil and animal faeces, including human excrement,^31^ are remarkably resilient, withstanding boiling, freezing and disinfectants, including ethanol.^32^ Inoculation may occur via farming or gardening injuries; in road traffic accidents; or falls, particularly with foreign body retention. Other routes include animal bites, burns, injections, and unclean surgical procedures, notably circumcision and termination of pregnancy.^33^ Once introduced, spores germinate, and bacilli secrete two key toxins: tetanolysin and TeNT. While the role of tetanolysin remains unclear, it may promote anaerobic conditions for bacterial proliferation.^34^

TeNT binds to peripheral motor neurons at the neuromuscular junction and undergoes retrograde axonal transport to the spinal cord and brainstem, similar to the rabies virus.^35,36^ Within the CNS, TeNT targets presynaptic glycine- and GABA-ergic interneurons, cleaving its substrate, synaptobrevin-2,^37^ and disrupting the SNARE complex essential for neurotransmitter release. The resultant failure of inhibitory neurotransmission leads to unchecked downstream motor activity, manifesting as muscle rigidity, spasms, and hyperreflexia. This also affects reflex circuits; unopposed motor activity within reflex arcs underlies stimulus-induced spasms of agonist and antagonist muscles. TeNT may spread through the lymphatics, bloodstream, and trans-synaptically within the CNS.^38,39^ Unlike TeNT, botulinum toxin type B—a closely-related neurotoxin that also targets synaptobrevin—acts peripherally without retrograde transport, and therefore engenders a dramatically different neurological syndrome characterised by rapidly-progressive descending flaccid paralysis, with a short incubation period.^37^ Autonomic dysfunction in severe tetanus is thought to be caused by disinhibition of autonomic neurons, resulting in dysregulated catecholamine release.^40^

Generalised tetanus, the most common and severe form of disease, typically progresses cephalocaudally. Cephalic and localised forms are predominantly determined by inoculation site (e.g. head/neck wounds, gingival infections, or chronic otitis media) and restricted toxin spread, respectively. It usually takes 4-6 weeks for inhibitory neurotransmission to be restored in generalised tetanus. The molecular mechanisms underlying protracted clinical recovery remain incompletely understood, but may include delayed clearance of cytosolic TeNT light chains, regeneration of synaptobrevin in pre-synaptic neurons and/or the sprouting of new axonal terminals.^41,42^

### History of Presenting Illness

While the notional incubation period for tetanus ranges from 1-60 days (median 8), symptom onset after 21 days is uncommon.^43^ Incubation is shorter for head and neck wounds but can be much longer with infected implants. In most cases, however, tetanus ensues from apparently innocuous or healed scratches and abrasions.^35^ Up to 30% of patients with tetanus have no identifiable wound on admission.^44–46^ Clinicians should therefore enquire about any injuries occurring in the prior 3 weeks, but maintain a high index of suspicion in any patient with compatible symptoms, particularly those with incomplete or unclear vaccination history. We suggest specifically asking about motorbike accidents,^47^ recent piercings, acupuncture, and subcutaneous and intramuscular injections, all of which are documented routes of infection.^48^

### Clinical Features

#### A) Source of Contamination

It is important to assess for occult wounds, as healed scratches or scabs may continue generating toxin. Physical examination should focus on the commonest inoculation site - the foot -^49^ and if there is a clear history, the body part where symptoms first started. Clinicians should elicit a history of injecting drug use and look for evidence of track marks and skin popping from subcutaneous opiate injection. Injecting drug use is also associated with poorer tetanus outcomes.^44,50^

Besides tetanus and its complications, clinicians should be alert to concurrent injury-related issues, notably skin and soft tissue infection, and consider the need for post-exposure rabies prophylaxis.^51^ Superinfections with leptospirosis from floodwater may layer jaundice upon features of tetanus, as is sometimes seen in Metro Manila during the rainy season. Finally, clinicians may encounter epiphenomena of alternative or traditional medicine treatments, such as linear scarring and annular skin changes from cutting needles and medicinal animal horns, respectively.

#### B) Clinical Presentation

**Figure 3:**
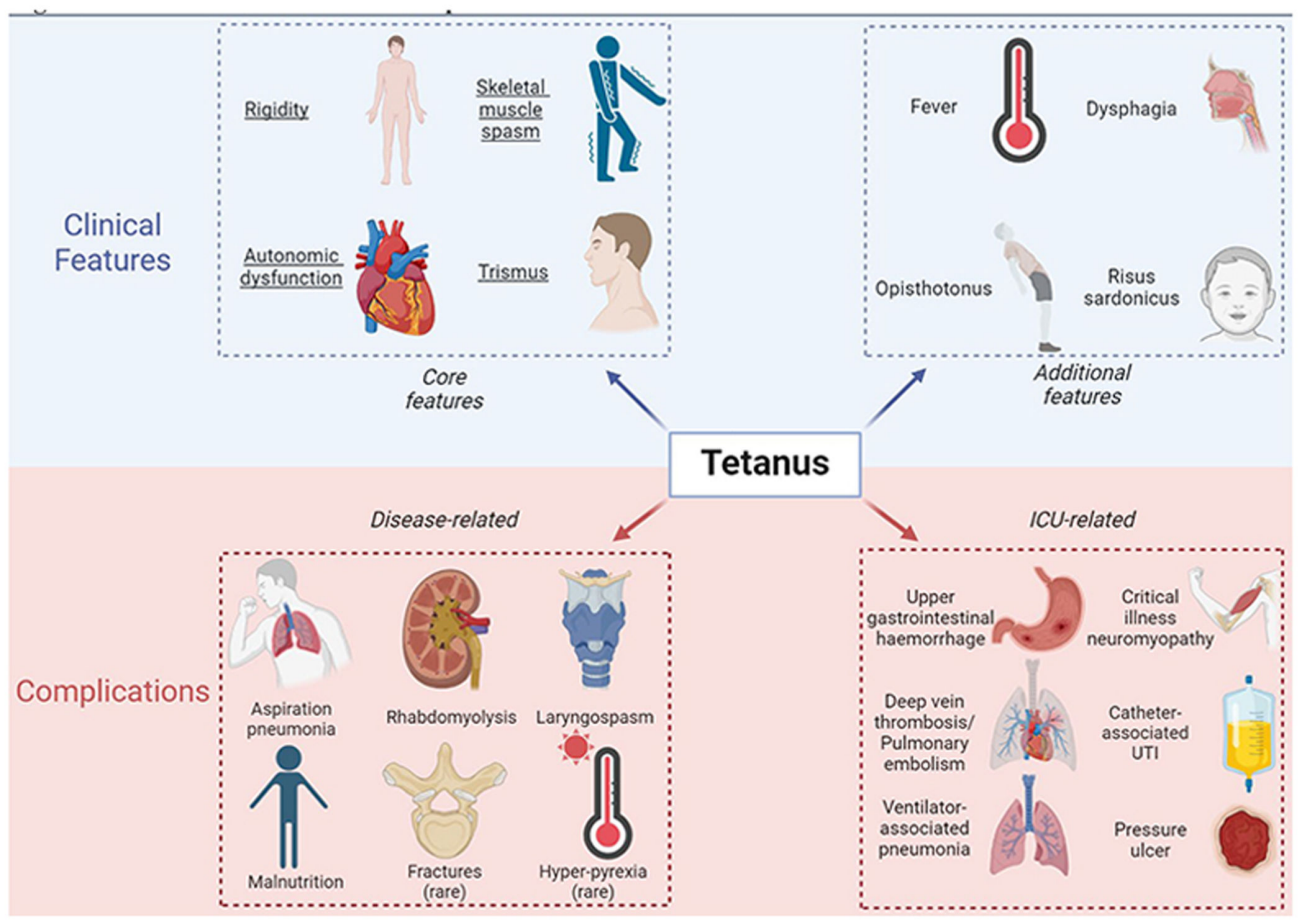
Clinical Features and Complications of Generalised Tetanus Legend: UTI = urinary tract infection. Figure created with Biorender.com.

Tetanus has a clinical spectrum of which the hallmarks are painful muscle spasms, and muscle stiffness from rigidity (Figure 3). Severity is usually categorised using the modified Ablett score.^52^ In Metro Manila, the commonest presenting symptoms of generalised tetanus are back and abdominal pain (due to truncal spasms), trismus and dysphagia.^53^ Voice changes may occur early in the disease course. Muscle rigidity - particularly abdominal rigidity - persists between spasms. This may mimic a peritonitic ‘acute abdomen’, and there are reported cases of unnecessary exploratory laparotomy in patients subsequently diagnosed with tetanus.^54^ Opisthotonus - extreme hyperextension of the back resulting in an accentuated arched posture - has long been regarded as pathognomonic of tetanus, but this is a relatively late clinical sign which is now encountered infrequently thanks to patients accessing treatment earlier in the disease course. Risus sardonicus is much more commonly seen at hospital admission, but is less specific for tetanus and should be interpreted within a wider clinical context.

Muscle spasms are exacerbated by stimuli, including loud noises, flashes of light, and touch, underscoring the importance of nursing patients in quiet, dark environments. Prolonged spasms may cause rhabdomyolysis, resulting in acute kidney injury (AKI). Spasms typically peak in the second week of illness, though, in our experience, older adults are at higher risk of early laryngeal spasm, even when limb spasms remain relatively mild. Patients may present with reduced oral intake, which may reflect trismus or dysphagia. Retrospective studies from the Ivory Coast and Vietnam suggest that trismus (93-98%) and dysphagia (83%) are almost always present at hospital admission.^55,56^ Severe dysphagia resulting from pharyngeal spasm confers a high risk of aspiration. Conversely, dyspnoea is rare at admission (5-10%), but may signal impending airway compromise and present a diagnostic challenge in resource-limited settings with suboptimal access to cross-sectional imaging. A few case reports have highlighted fractures due to severe spasms,^57^ but in our experience, this is very rare.

Typical neurological examination findings in generalised tetanus include bilateral hypertonia with superimposed spasms (reflecting simultaneous agonist-antagonist muscle contraction), hyperreflexia, and reduced power. Sensory and cerebellar examination is usually normal. Unlike many neurological infections, lucidity is preserved. Obtundation should prompt consideration of differential diagnoses such as neuroleptic malignant syndrome (NMS), or related epiphenomena, such as opioid misuse.

Autonomic nervous system dysfunction (ANSD) has become the most feared complication of tetanus. Dysautonomia has protean clinical manifestations, is difficult to manage, and confers a poor prognosis. Historically, this was thought to arise 1-2 weeks after symptom onset.^40^ However, recent studies, employing sensitive, wearable digital devices, suggest that autonomic dysfunction is often present at disease onset in neonates, and develops within short days in adults.^58^ Features of dysautonomia include painful acute urinary retention; faecal incontinence; and profuse sweating. Cardiovascular instability is seen in 10-30% patients and manifests as bouts of tachycardia and hypertension, lasting minutes to hours, that often alternate with spells of bradycardia and profound hypotension.^59,60^ Fever >38.4°C is relatively rare at first presentation (in one Vietnamese cohort, it was identified in just 8% patients at diagnosis)^61^ and often signals superadded infection or a distinct diagnosis like NMS. In patients with dysautonomia, however, fever may persist for weeks. This may contribute to rhabdomyolysis, and confound clinicians assessing for concurrent hospital-acquired infection.

Localised tetanus is rare, and presents with muscle spasms and rigidity localised to the area around the site of injury, usually a single limb. It is more common in partially immune individuals, such as those who have had primary childhood immunisation but no subsequent boosters. Diagnosis is challenging, but prognosis comparatively favourable, despite the risk of secondary generalisation.^62^

Cephalic tetanus, a variant of localised tetanus, is confined to the head and neck. Such patients may present to stroke services, with facial palsies, dysphagia and dysarthria.^63^ Symptoms often fluctuate, leading to a misdiagnosis of transient ischaemic attack. Both flaccid and spastic paralysis can occur in cephalic tetanus, sometimes concurrently.^64,65^ This confusing presentation reflects dual sites of TeNT action. Historic electrophysiological studies and recent rodent experiments suggest that TeNT acts peripherally at facial neuromuscular junctions and centrally at the facial nucleus, giving rise to flaccid and spastic weakness, respectively, which may be uni- or bilateral.^65,66^ Neuroimaging will not identify acute parenchymal abnormalities, though the presence of gas locules may add weight to a probable diagnosis.^63^ Cephalic tetanus may generalise without prompt recognition, and it should be considered when patients present with one or more cranial nerve palsies alongside trismus and/or risus sardonicus. Careful examination of the scalp may reveal a contaminated wound.

### Diagnosis

Tetanus is a clinical diagnosis. Specific diagnostic tests, such as rapid immunoassays, lack standalone confirmatory utility, and may be inaccessible in resource-constrained settings where tetanus is most common.^67,68^ Local or national case definitions aid early recognition and treatment. At the Hospital for Tropical Diseases, Ho Chi Minh City, Vietnam, tetanus is defined as ‘presence of trismus, dysphagia and continuous generalised muscle rigidity or spasms, in the presence of a normal conscious level and without fever at onset.’ In contrast, the UK Health Security Agency defines tetanus as an ‘acute illness with muscle spasms or hypertonia, and a diagnosis of tetanus made by a healthcare provider.’^13^

The differential diagnosis for generalised tetanus (Table 1) includes hypocalcaemic tetany, NMS, drug-induced dystonias, progressive encephalomyelitis with rigidity and myoclonus, stiff-person syndrome, cerebral malaria and strychnine poisoning. Although banned across the European Union and Canada, strychnine-based rat poison is widely available in the USA and many low- and middle-income countries. Strychnine, a competitive glycine receptor antagonist, produces a clinical syndrome virtually indistinguishable from tetanus when accidentally or deliberately consumed.^69^ Cephalic tetanus may mimic brainstem stroke, Bell’s palsy, myasthenia gravis, or botulism.

Basic tests help rule out differentials and identify complications. All suspected cases should undergo urinalysis to assess for haematuria (a proxy for myoglobinuria) to exclude spasm-induced rhabdomyolysis alongside serum creatine kinase (CK) level where available. Urine is also the preferred sample type for suspected strychnine poisoning.

Other blood tests, including serial full blood count, C-reactive protein (CRP), renal, liver and bone profiles, magnesium, and blood gas analysis are also useful. Mild leukocytosis is common; neutrophilia is a poor prognostic indicator.^56^ CRP is usually moderately elevated, but may be normal; a marked rise may signify concurrent bacterial infection (typically cellulitis or sepsis at first presentation, and nosocomial infection later). Renal profile may identify AKI, bone profile can exclude hypocalcaemic tetany, and serum magnesium monitoring is useful during IV replacement.

The spatula test,^70^ though sensitive and specific, is no longer recommended due to the risk of precipitating laryngospasm and airway obstruction. Wound imaging by CT may identify foreign bodies or gas locules. Neuro-imaging or lumbar puncture are usually unnecessary.

Supplementary tests (Table 2), can play a supportive role in diagnosis. The microbiological tests of choice are real-time polymerase chain reaction (RT-PCR) for *C. tetani* neurotoxin gene detection, and *C. tetani* culture. RT-PCR is usually favoured for its faster turnaround time and ability to confirm toxigenic, disease-causing strains. The optimal sample is tissue obtained at surgical debridement, transported in an anaerobic medium such as cooked meat broth. Quantitative serological testing for TeNT antibodies to evaluate pre-existing immunity in suspected tetanus was previously common, but cases have occurred in immunised patients despite ‘protective’ antibody titres.^71,72^ This test is therefore falling out of favour in countries such as the UK. If performed, it is crucial that serum samples be taken prior to immunoglobulin administration. All these tests are useful when positive in symptomatic patients, but, crucially, negative results cannot exclude tetanus. Unless advised to do so by an expert microbiologist, we would counsel against testing asymptomatic patients.

### Management

Management is multifaceted and guided by disease severity. Although useful for early prognostication, the Ablett classification is confounded by subjective grading and patient comorbidities (e.g. concurrent bacterial infection). As such, clinicians should apply their clinical judgement and consider early ICU admission even for patients with ‘mild’ disease.

Core management principles include source control and antibiotic administration; neutralising unbound toxin; airway management; spasm control; managing dysautonomia; and high-quality supportive care. Importantly, natural infection does not confer immunity, so all patients who recover from tetanus require a full course of immunisation.

### Halting toxin production

Surgical debridement is required to eradicate necrotic tissue that may harbour *C. tetani*, even for wounds that appear innocuous. Debridement serves the dual benefit of providing samples for microbiological testing. For inoperable wounds, scrupulous wound care is key. Enteral or intravenous (IV) antibiotics should be administered for 7-10 days. Few studies have compared metronidazole (an inexpensive drug with excellent anaerobic coverage) and penicillin (more widely available, but with higher risks of allergic reactions). The first open-label trial of these drugs, published in 1985, found a significantly lower risk of death in Indonesian patients who received metronidazole.^73^ However, 3 subsequent studies failed to corroborate any difference in mortality, length-of-stay, or need for MV.^74–76^ Yen and colleagues did find a lower muscle relaxant requirement in the metronidazole group,^76^ which may reflect theoretical effects of penicillin on GABAergic neurotransmission.^77^ As such, the evidence base marginally favours metronidazole, but penicillin is an effective, well-tolerated alternative.

### Neutralising unbound toxin

Administration of antitoxin via an intramuscular (IM) or IV route improves mortality in tetanus. Antitoxin is derived from horses or pooled plasma provided by fully vaccinated human blood donors. Historically, human tetanus immunoglobulin (hTIG) has been preferred due to concerns about adverse reactions (anaphylaxis and serum sickness) with equine antitoxin, but its high cost and global shortages have made the latter the standard of care in many high-burden, resource-constrained settings.^78^

A recent trial in Vietnam found no difference in mortality, MV duration, or ICU/hospital stay between patients randomised to equine antitoxin and hTIG. Adverse events were also rare in both groups.^79^ Nonetheless, the full equine antitoxin dose should be administered only after a negative IM test dose. Adult dosing practices vary widely (500-10,000 IU); further research to identify optimal regimens may help to preserve limited supplies.

hTIG shortages have prompted some countries to switch to intravenous immunoglobulin (IVIG). This contains unfractionated immunoglobulins rich in tetanus immunoglobulin thanks to widespread vaccine uptake. In the UK, hTIG is reserved for post-exposure prophylaxis in unprotected patients with tetanus-prone wounds. Patients with confirmed tetanus are instead treated with IVIG, with weight-based dosing equal to 5000 IU (<50kg patient) or 10,000 IU (>50kg patient) of anti-tetanus antibodies.^13^ Given the varied composition of commercially available IVIG products, this equates to 200-800ml, at an NHS cost exceeding £2,000 per patient. Monoclonal antibodies have shown promise in preclinical studies,^80,81^ and the positive results of a phase III trial of prophylactic siltartoxutag following tetanus-prone exposures were recently presented at a research meeting of the American College of Emergency Physicians.^82^ However, monoclonal antibodies are likely to remain out-of-reach in high-burden, resource-constrained settings, due to high cost and cold-chain requirements.

Several low-quality trials have reported positive clinical outcomes with intrathecal (IT) antitoxin administration.^83–85^ However, a robust trial involving 272 Vietnamese adults found no significant differences in need for MV or all-cause mortality at 6 months when IT antitoxin was added to IM therapy.^79^ While IT administration was not associated with significant enduring adverse effects, it carries theoretical risks of CNS infection and may precipitate spasms. Given the unclear benefit, we do not recommend IT antitoxin administration based on current evidence.

### Airway Management

Across all settings, around 50% of adults with generalised tetanus require MV.^86–88^ Though many patients on first presentation have a small oxygen requirement and difficulty clearing secretions, the primary indication for MV is protecting the airway from fulminant obstruction precipitated by laryngeal spasm. MV is generally recommended for all Ablett III/IV cases. Where available, primary tracheostomy is preferred, as prolonged endotracheal intubation carries increased risks of subglottic tracheal stenosis and vocal cord immobility. Percutaneous tracheostomy is preferred in some settings to minimise spasms provoked by operating theatre transfer. Maintaining consistent access to primary tracheostomy, particularly out of hours, may be challenging. In our experience, fostering strong relationships with Ear, Nose and Throat (ENT) surgeons and intensivists ensures that patients access timely, optimal ventilatory support.

### Control of Muscle Spasms

Benzodiazepines are the cornerstone of spasm control. Both diazepam and midazolam are widely used, but have never been compared head-to-head. Diazepam is cheaper, and can be administered by various routes, but its long-acting metabolites can persist for days, conveying greater risks of respiratory depression, especially in patients with renal impairment. High doses, often >1mg/kg/day of diazepam, are usually required. Protocols vary, but many escalate from initial IV boluses to continuous infusion, before switching to midazolam.

If benzodiazepines prove inadequate for control, most guidelines advocate for neuromuscular blockade (NMB). As these agents cause paralysis, they are only safe in ventilated, sedated patients. No single NMB agent is optimal; vecuronium, pipecuronium and rocuronium, being cardiovascularly inert, are generally preferred where available.^46^ Propofol is occasionally used as an adjunct for sedation and spasm control. IV magnesium sulphate is discussed below.

Other drugs including barbiturates, phenothiazines, and dantrolene sodium, are rarely used unless first-line medications are unavailable. Intrathecal baclofen has been used for refractory spasms, but is not widely practised given procedural risks and mortality concerns.^89^

### Management of Dysautonomia

The mainstays of treatment for dysautonomia are IV magnesium sulphate and opioids. Short-acting opiates, such as fentanyl and morphine, provide analgesia, sedation, and autonomic control, though high doses may be impractical in settings where opioid availability is low.

A recent systematic review involving 13 studies concluded that IV magnesium sulphate may improve dysautonomia control and shorten hospital stays.^90^ One robust double-blind trial, published in 2006, reported reduced mean heart rate and need for midazolam and pipecuronium among 195 Vietnamese adults randomised to 7 days of IV magnesium or placebo.^91^ However, this did not translate into statistically significant differences in the need for MV, duration of hospitalisation, or in-hospital mortality. Nevertheless, magnesium sulphate remains widely used and in our experience, it is a safe and effective drug. We recommend an initial bolus followed by continuous infusion, for up to one week, targeting serum levels of 2-4mmol/l. In resource-constrained settings, magnesium therapy can be titrated to knee jerk at the bedside in lieu of frequent blood tests.

Patients with tachycardia and hypertension frequently require treatment with adrenoceptor antagonists. Labetalol, a combined alpha and beta adrenoceptor blocker, is often preferred due to its short half-life. Though evidence is limited, clonidine, dexmedetomidine and verapamil are also helpful in our experience. Inotropes may be necessary for refractory shock, and atropine for bradyarrhythmias. This lability presents practical challenges for nurses, who may have to manage multiple competing infusions in critically ill patients.

### General supportive care

Prolonged immobility confers increased risks of pressure area breakdown and venous thromboembolism (VTE). Where available, air mattresses should be used, and in low-resource settings with nursing shortages, family members should be taught how to turn patients safely.

Judicious nursing, balancing the need for pressure area care against spasm-inducing tactile stimulation, is key. Relatives should be advised to avoid excessive physical contact at the bedside. Intermittent pneumatic compression stockings should be avoided as they trigger spasms; pharmacological VTE prophylaxis is preferred. Stress ulcer prophylaxis should be administered to ventilated patients unless contraindicated.^92^

At first presentation, many patients are severely dehydrated due to poor oral intake, later exacerbated by insensible losses from hyperpyrexia. Fluid resuscitation is often necessary, followed by maintenance of euvolaemia. Most patients require a urinary catheter for bladder stasis and to facilitate accurate fluid balance monitoring. Poor oral intake also increases the risk of hypoglycaemia; measuring blood glucose levels is therefore important in the acute phase. Patients with hyperpyrexia benefit from passive cooling and regular anti-pyretics. Generalised spasms, when uncontrolled, result in marked energy expenditure and thus high nutritional requirements. Even in patients maintaining their own airway, we recommend early feeding via a nasogastric tube, to reduce the risk of aspiration. Rehabilitation efforts, including passive limb mobilisation, should be initiated early to prevent contractures, but active physiotherapy should be deferred until spasms have abated.

**Figure 4:**
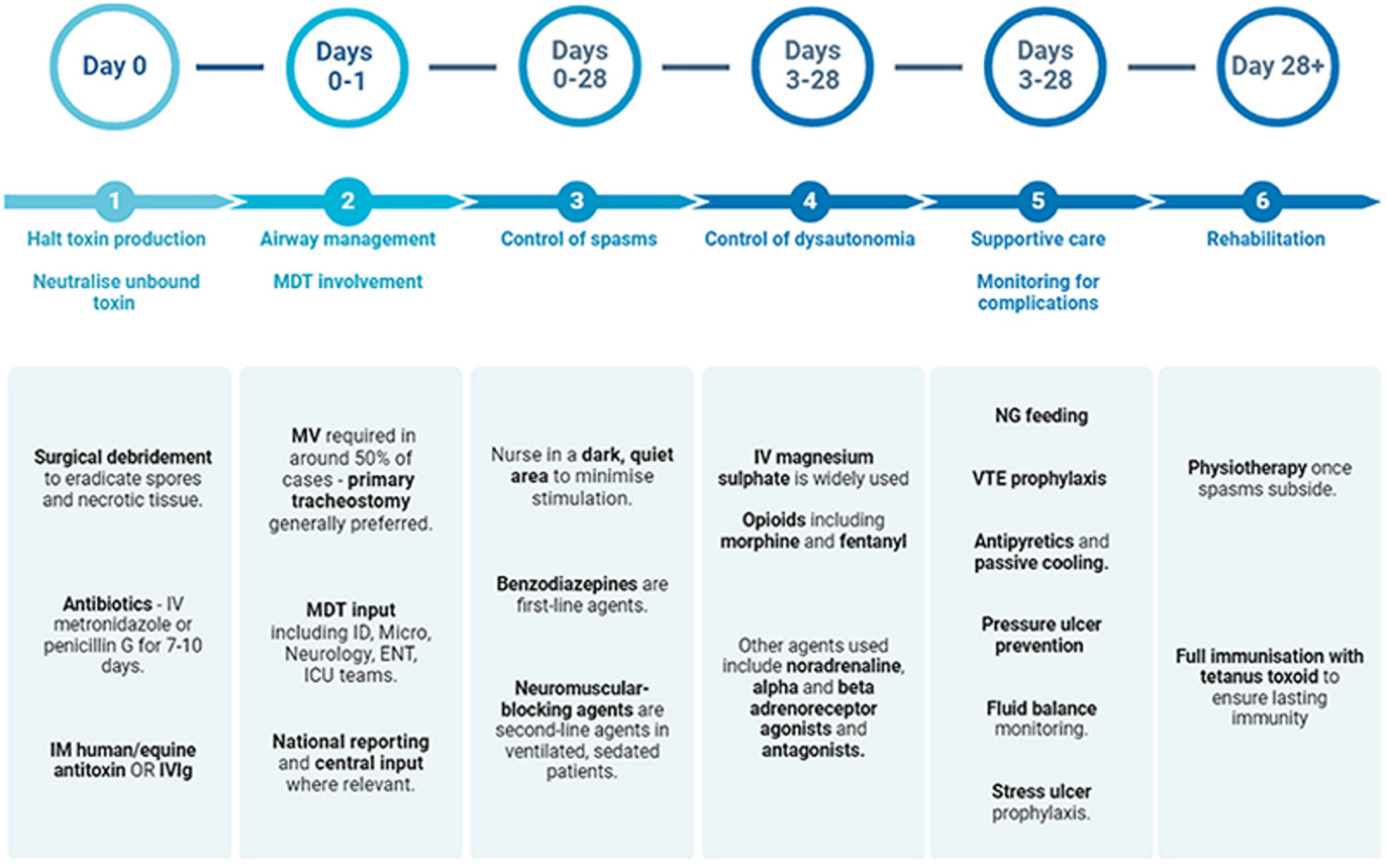
Tetanus management principles and workflow Legend: IV = intravenous; IM = intramuscular; IVIg = intravenous immunoglobulin; MV = mechanical ventilation; MDT = Multi-Disciplinary Team; ID = Infectious Diseases; Micro = Microbiology; ENT = Ear, Nose & Throat; ICU = Intensive Care Unit; NG = nasogastric; VTE = venous thromboembolism. Figure created with Biorender.com.

Figure 4 outlines a tetanus management timeline.

### Prognosis

Adult tetanus case fatality rates (CFRs) vary widely (5-50%), reflecting disparities in care access. Historically, respiratory failure due to laryngeal spasm was the leading cause of death in tetanus. Access to ventilatory support has improved outcomes and shifted mortality patterns in well-resourced settings, where cardiovascular events - most commonly arrhythmias from ANSD - are now the prevailing cause of death, particularly in elderly patients.^44,93,94^ The lowest reported CFR worldwide - 2.4% - likely reflects ready MV access, clear treatment protocols and intensivist expertise in a higher-prevalence Vietnamese setting.^87^ The median MV duration in this cohort was 16 days.

Several studies characterising risk factors for tetanus mortality were conducted prior to widespread access to ventilatory support, limiting their contemporary relevance and usefulness. Nonetheless, extremes of age, immunocompromise, and generalised tetanus are associated with worse prognosis. Other negative prognostic indicators include short (<7 day) incubation period, short interval between first symptom and first spasm, short time from first symptom to hospitalisation, high Ablett score, ANSD, and need for MV.^75,95–98^

Much of the remaining mortality and morbidity from tetanus is indirect and stems from complications of ICU care. These include nosocomial infections, stress ulcers, VTE, pressure sores and critical care neuromyopathy.^97,98^

Long-term outcomes in tetanus survivors remain understudied. One Japanese case series found that 175/499 (35%) survivors were discharged to non-home settings.^86^ In an older French cohort, 61% of ICU survivors had no lasting disability after a median follow-up of almost 4 years.^99^ These findings highlight good recovery potential, even in elderly, comorbid patients. We encourage intensivists battling ICU capacity constraints to persist with continued care - recovery is slow, but most patients improve significantly with time and supportive treatment.

Lastly, prolonged intensive care can be financially ruinous. In many high-burden settings, the poorest people face the greatest risk of acquiring tetanus, presenting late, and requiring ICU admission. Education, universal health coverage, and horizontal health system strengthening are essential to reduce these disparities. However, out-of-pocket expenses remain high even where coverage exists;^100^ in one South Korean study, healthcare costs exceeded $18,000 (approximately £14,000).^88^

### Conclusion

Tetanus remains a severe disease with substantial mortality and global burden. Clinicians should maintain a high index of suspicion in patients presenting with trismus, dysphagia, rigidity or spasms. High CFRs in resource-limited settings underscore the need for early diagnosis and access to high-quality critical care sustained over long periods. Tetanus can be prevented with timely wound care and by strengthening vaccination programmes. Further research is needed to optimise antitoxin dosage and routes of administration; identify tractable biomarkers to assist in early diagnosis and prognostication; and rehabilitate critical care survivors.^101^

## Figures and Tables

**Table 1:** Differential Diagnosis of Generalised Tetanus with Overlapping and Discerning Features

Differential Diagnosis	Overlapping Features	Discerning Features
**Dental infection**	Trismus	History of toothache or dental pathology
	Dysphagia	Presence of dental abscess
	Fever*	Normal muscle tone and absence of spasms
**Strychnine poisoning**	Clinical syndrome virtually indistinguishable from generalised tetanus	History of accidental or deliberate exposure to strychnine, typically as rat poison
	··	Rapid onset within 30 minutes of ingestion, inhalation, or intravenous exposure, or within 12 hours of dermal exposure
	··	Positive assay on serum, urine, or gastric aspirate
**Drug-induced dystonia**	Muscle rigidity	History of recent exposure to antidopaminergic or serotonergic agents
	Trismus	Nature of involuntary movements e.g. ocular deviation not seen in tetanus
	Muscle spasms	Absence of tonic muscle contraction between spasms
	··	Reversible with anticholinergic agents such as procyclidine
**Neuroleptic malignant syndrome**	Muscle rigidity	History of exposure to antipsychotic agents
	Autonomic nervous system dysfunction - notably hyperpyrexia*	Gradual onset
	··	Altered mental status
	··	Very high creatine kinase (CK) levels
**Hypocalcaemia (tetany)**	Muscle spasms	Presence of risk factors for hypocalcaemia
	Laryngospasm	May additionally cause parasthesiae
	Cardiac dysrhythmias	Altered mental status, seizures, or movement disorders
	··	Absence of trismus
	··	Low serum calcium on blood test
**PERM / Stiff-person syndrome**	Muscle rigidity	Subacute progression over weeks
	Spasms of trunk and limbs precipitated by voluntary movement or auditory/tactile/emotional stimuli	Brainstem signs e.g. ophthalmoplegia, cerebellar ataxia
	Hyperreflexia	Absence of trismus or facial spasms
	··	Rapid response to diazepam
	··	Positive GAD and anti-glycine antibodies
**Cerebral malaria**	Fever*	History of travel to endemic areas
	Opisthotonus (especially in children and adolescents)	Altered mental status and seizures common
	··	Absence of trismus and risus sardonicus
	··	Presence of parasitaemia on blood smear.
**Rabies**	Fever*	Longer incubation period (typically 2-3 months)
	History of animal bite or scratch	Hydrophobia and aerophobia
	Presence of inducible spasms, typically pharyngeal	Rapid progression to encephalopathy with altered consciousness

Legend: PERM = Progressive Encephalomyelitis with Rigidity and Myoclonus.

* Fever may occur in tetanus, particularly in patients with dysautonomia, but early, high-grade fevers should prompt consideration of differential diagnoses or superimposed infection.

**Table 2:** Diagnostic Tests in Tetanus

Diagnostic Test	Results Supportive of Tetanus Diagnosis	Advantages	Disadvantages and Pitfalls	Recommendations
**PCR for *C. tetani***	**Detection of *C. tetani* toxin gene from debrided tissue or pus sample**	- Most sensitive test currently in use	- Limited global availability − generally only in reference laboratories	Where available, this is the best modality of diagnosis: perform in all patients with suspected tetanus
	··	- Rapid turnaround time if on-site facilities available	- False negatives still possible depending on sample quality	- Positive results are supportive in presence of the clinical syndrome
	··	- Tissue debridement is part of standard tetanus care	- Not suitable for all patients - wound site for tissue sample may not be identifiable	- Negative results do not exclude diagnosis
**Wound culture**	**Growth of C. tetani from anaerobic culture of debrided tissue or pus**	- High specificity and positive predictive value in symptomatic patients	- Fastidious organism - can be challenging to culture	Where available, perform on pus or debrided tissue in all patients with suspected tetanus
	··	- Tissue debridement is part of standard tetanus care	- Not suitable for all patients - wound site for tissue sample may not be identifiable	- Positive results are diagnostic in presence of the clinical syndrome
	··	- Identifies any co- incident or superimposed pathogens in wound	- Limited sensitivity - negative culture does not exclude tetanus	- Negative results do not exclude diagnosis
	··	··	- Diagnostic delay due to culture time	··
	··	··	- Toxigenic *C. tetani* can be isolated from wounds in patients without clinical tetanus	··
	··	··	- Isolation of *C. tetani* does not imply pathogenicity (strain may lack plasmid encoding TeNT)	··
**Serology for Anti- TeNT Antibody Levels**	**Low (<0.1 lU/ml) titre of protective antibodies in a patient with a compatible clinical syndrome**	- Sub-protective levels of anti- tetanus antibodies are supportive of diagnosis	- Antibody levels above 0.1 lU/mL do not exclude diagnosis	Where available, consider in all patients with suspected tetanus
	··	- No tissue or pus sample required	- Treatment with antitoxin/IVIg prior to taking blood test may confound results	- Serum sample must be taken prior to antitoxin/IVIg administration
	··	- Determines immunity status, informing revaccination needs	- High titres in symptomatic patients may represent seroconversion and give false reassurance	- Positive results are diagnostic in presence of the clinical syndrome
	··	··	··	- High antibody titres do not exclude diagnosis
**Serum tetanus toxin bioassay**	**Presence of tetanus toxin in serum - confirmed by inoculation into animal (usually mouse)**	- Confirms presence of active toxin, providing definitive evidence of infection due to high specificity	- Limited global availability − generally only in reference laboratories	Consider in patients with suspected tetanus as per national guidance for confirmatory testing
	··	- No tissue or pus sample required	- Little data regarding sensitivity, particularly in localised/cephalic tetanus − negative results cannot exclude tetanus	- Positive results are diagnostic in presence of clinical syndrome
	··	··	- Ethical concerns due to animal use	- Negative results do not exclude diagnosis
**Electromyography / Nerve Conduction Studies**	**Spontaneous & asynchronous activation of motor units firing at a rate of 4-15 Hz, with limited voluntary control and a shortened (or absent) silent period**	- No tissue or pus sample required	- Pain invoked during nerve conduction studies can precipitate severe spasm	Consider where available in patients for whom diagnostic uncertainty exists

## What was known before?

Deaths due to neonatal tetanus have fallen precipitously in recent decades, thanks to significant improvements in access to vaccination. Nonetheless, adult tetanus remains a major problem, particularly in low- and middle-income settings with restricted access to ventilatory and circulatory support. Tetanus has a global distribution, and, although rare in high-income countries with high vaccination coverage, it is associated with significant morbidity and mortality, often due to diagnostic delays. While sustained intensive care—focusing on airway management, spasm control, and dysautonomia—can achieve good clinical outcomes, emerging evidence continues to refine diagnosis and clinical practice.

## Search strategy

We identified references for this review by searching PubMed, Medline, and Cochrane databases, with no language or date restrictions, up to 18^*th*^ November 2024. Search terms incorporated Boolean operators and included “tetanus”, “diagnos* OR test OR investigat*” and “treat* OR manage*”. Additional references were identified by reviewing citation lists from relevant articles and textbook chapters. Publications from the last five years were selected preferentially, though some older studies were included where they were widely cited or particularly influential for clinical practice.

## Key Points

1. The hallmarks of tetanus are muscle spasms and rigidity; suggestive clinical features include trismus, risus sardonicus, and abdominal and back pain
2. In settings with high vaccination coverage, risk factors include older age, male sex, domestic/gardening injuries, immunosuppression, and injecting drug use
3. Tetanus is primarily a clinical diagnosis, though culture and molecular testing of pus or debrided tissue can provide supportive information
4. Key management principles include securing the airway; preventing further toxin production and neutralising circulating toxin through wound debridement, antibiotic therapy, and antitoxin; and managing painful muscle spasms/dysautonomia
5. Perseverance is crucial: tetanus can have a protracted course, but most patients can achieve full recovery with time and sustained ventilatory support.
6. Tetanus does not confer immunity against future infection; after recovery, all patients require a full course of immunisation.
